# The Ratio of Visceral to Subcutaneous Adipose Tissue Is Associated With Postoperative Anastomotic Leakage in Patients With Rectal Cancer With Gender Differences in Opposite Direction

**DOI:** 10.1002/cam4.70933

**Published:** 2025-05-09

**Authors:** Yan Luo, Jian Liu, Jiong Huang, Liya Ma, Zhen Li

**Affiliations:** ^1^ Department of Radiology Tongji Hospital, Tongji Medical College, Huazhong University of Science and Technology Wuhan China; ^2^ Department of Radiology Wuhan Hospital of Traditional Chinese Medicine Wuhan China; ^3^ Department of Radiology The Sixth Hospital of Wuhan, Affiliated Hospital of Jianghan University Wuhan China

**Keywords:** anastomotic leakage, computed tomography, ratio of visceral to subcutaneous adipose tissue, rectal cancer

## Abstract

**Background:**

Anastomotic leakage (AL) is a severe postoperative complication in colorectal cancer and exerts negative impacts on patients' outcomes. Studies have found that body composition measured by CT images was associated with increased overall postoperative complications in colorectal cancer; however, few focused on postoperative AL in rectal cancer. This study aimed to explore the association between body composition parameters measured by CT images and postoperative AL in patients with rectal cancer, with an emphasis on subgroup analysis by gender.

**Methods:**

From February 2014 to January 2020, a total of 444 patients with rectal adenocarcinoma who underwent radical proctectomy were included. Out of all patients, 21 developed AL after surgery. Body composition parameters, including the areas, mean CT values, height‐normalized indices of subcutaneous adipose tissue (SAT), visceral adipose tissue (VAT), intramuscular adipose tissue (IMAT) and skeletal muscle (SM) were derived from preoperative contrast‐enhanced arterial phase CT images at the third lumbar level. The ratio of visceral to subcutaneous adipose tissue (VSR) was calculated. Clinical and body composition parameters were compared between the AL group and the non‐AL group in all patients and separately in different genders.

**Results:**

Body composition parameters were not significantly different in the AL group and the non‐AL group in all patients. However, most body composition parameters were significantly different between male and female patients. After separately analyzing by gender, VSR was significantly associated with postoperative AL in male and female. After multivariate regression, VSR remained an independent predictor for AL (OR: 0.1, *p* = 0.041 for male and OR: 39.1, *p* = 0.045 for female).

**Conclusion:**

The VSR measured by CT images is an independent predictor for postoperative AL in patients with rectal cancer; however, it shows gender differences in opposite directions, serving as a protective factor in males, whereas as a risk factor in females.

## 1 Introduction

Obesity characterized by the excessive accumulation of adipose tissue poses a major risk for many other diseases and health problems, such as metabolic syndrome and cancers [1]. Body mass index (BMI), defined as weight in kilograms divided by the square of height in meters, is a common index to assess the severity of obesity; however, it fails to reflect body composition [2]. In recent years, lots of studies have indicated that body composition, which can be measured by CT images, is better associated with the level of health risk than BMI [3]. Besides obesity, CT images can also evaluate other abnormal body composition phenotypes such as myosteatosis (abnormal fat accumulation in the muscles) and sarcopenia (muscle loss) [4, 5].

Colorectal cancer ranks as the third most common cancer and the second most deadly cancer worldwide in 2020 [6]. Anastomotic leakage (AL) is a severe postoperative complication in colorectal cancer with a reported incidence of 5%–19% [7]. AL leads to increased postoperative morbidities and mortalities, prolonged hospitalization, and exerts negative impacts on patients' survival. Many researchers have investigated potential risk factors for AL, such as male gender, obesity, comorbidities, tumor location, and type of surgical approach and procedure [8]. Identifying risk factors for AL allows for better prevention. Recently, studies have found that body composition measured by CT images was associated with increased overall postoperative complications in colorectal cancer [9, 10]; however, few focused on postoperative AL in rectal cancer.

Therefore, this study aimed to explore the association between body composition parameters measured by CT images and postoperative AL in patients with rectal cancer. Given the gender differences in body composition [11], we especially placed an emphasis on subgroup analysis by gender.

## 2 Materials and Methods

### 2.1 Patients Selection

This retrospective study was approved by our institutional review board (approval number: TJ‐IRB202420206) and informed consent was waived. Between February 2014 and January 2020, newly diagnosed rectal adenocarcinoma patients who underwent radical proctectomy were reviewed from the electronic medical record (EMR) system.

Inclusion criteria were as follows: (1) Aged ≥ 18 years (2) pathologically diagnosed with rectal adenocarcinoma; (3) underwent abdominal CT examination before surgery within 4 weeks; (4) with or without neoadjuvant therapy. Exclusion criteria were as follows: (1) without pre‐surgical abdominal CT examination; (2) incomplete clinical or imaging data.

Demographic characteristics and medical history were extracted from EMR. Demographic characteristics included sex, age, height, and body weight of each patient. BMI was calculated and classified into the following four categories: < 20.0 kg/m^2^, underweight; 20.0–24.9 kg/m^2^, normal; 25.0–29.9 kg/m^2^, overweight; ⩾ 30.0 kg/m^2^, obese. Medical history included comorbidities, such as diabetes, hypertension, and cardiovascular diseases, daily alcohol consumption and duration time, and daily cigarette intake and duration time. The smoking index was calculated by multiplying the number of cigarettes smoked per day by the number of years of smoking. The results of laboratory tests were recorded, including the count of white blood cells (WBC) and neutrophils, C‐reactive protein (CRP), procalcitonin (PCT), triglycerides (TG), cholesterol (CHOL), albumin (ALB), hemoglobin (Hb) before surgery within 30 days, and WBC and neutrophils after surgery within 5 days.

The details of surgical procedure were processed into categorical variables, for example, surgical procedure method (anterior resection, low anterior resection, very low anterior resection or other method), whether performed ileostomy, whether performed laparoscopy or not, tumor location (upper, middle or lower segment of rectum), the tumor size (cm), the TNM stage (according to the 8th edition of the American Joint Committee on Cancer [AJCC]'s cancer staging manual) [12], the clinical stage and the American Society of Anesthesiology (ASA) score [13]. Postoperative AL was diagnosed by surgeons based on radiological and clinical findings [14]. Radiological findings include discontinuity of the bowel wall of the anastomosis with nearby gas or fluid collections on CT images or extraluminal contrast extravasation on water‐soluble contrast enema containing iodine. Clinical findings include fecal, feculent or purulent intraperitoneal drainage and positive digital rectal examination [14, 15]. In some patients the diagnosis of AL was further confirmed by relaparotomy. The severity of AL was graded according to the Clavien‐Dindo classification of surgical complications [16].

### 2.2 CT Image Analysis

**FIGURE 1:**
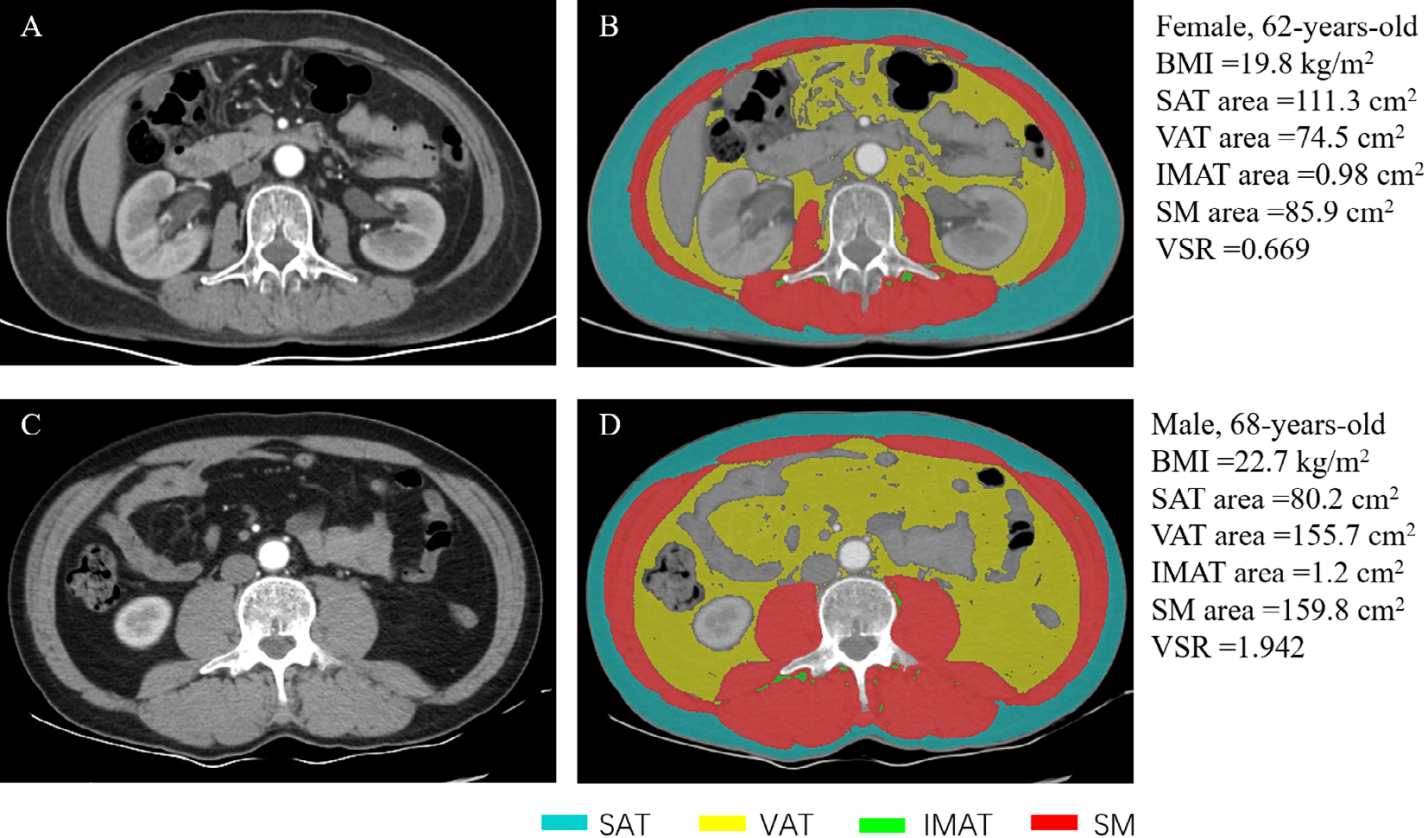
Cross‐sectional CT images at the third lumbar vertebra level used to measure body composition variables. BMI, body mass index; IMAT, intramuscular adipose tissue; SAT, subcutaneous adipose tissue; SM, skeletal muscle; VAT, visceral adipose tissue. (A, B) Female, 62 years old; (C, D) male, 68 years old.

SliceOsmatic 5.0 software (TomoVision, Magog, Canada) was applied to measure body composition of each patient. The axial contrast‐enhanced arterial phase CT image at the third lumbar vertebra (L3) level in DICOM format was imported into the SliceOsmatic. The software then semi‐automatically demarcated adipose tissue and skeletal muscle based on predetermined Hounsfield unit (HU) thresholds as previously described: −190 HU to −30 HU for subcutaneous adipose tissue (SAT) and intramuscular adipose tissue (IMAT), −150 HU to −50 HU for visceral adipose tissue (VAT), and −29 HU to 150 HU for skeletal muscle (SM, including internal and external oblique muscles, transversus abdominis, psoas, paraspinal and rectus abdominis) [17, 18]. Representative images are shown in Figure 1. The areas and mean CT values of SAT, VAT, IMAT, and SM were obtained. The area of SAT, VAT, IMAT, and SM in square centimeters was normalized for the square of height in meters to calculate SAT index (SATI), VAT index (VATI), IMAT index (IMATI) and SM index (SMI), respectively. The ratio of visceral to subcutaneous adipose tissue (VSR) was also calculated by the area of VAT divided by the area of SAT [17].

### 2.3 Statistical Analysis

Statistical analysis was performed using SPSS 22.0 (SPSS Inc., Chicago, IL). Categorical variables are expressed as numbers (percentages). Continuous variables are expressed as mean ± standard deviations or median (interquartile ranges). Chi‐squared test or Fisher exact test was applied for categorical variables. For continuous variables, if normally distributed, the student‐*t* test was applied; if not normally distributed, the Mann–Whitney *U* test was applied. Univariate and multivariate logistic regression analyses were performed to identify independent predictive variables for AL. Since previous studies revealed that the body composition of adipose tissue and skeletal muscle was significantly different between female and male individuals [11], our study analyzed the data separately by gender. Receiver operating characteristic (ROC) curve analysis was performed to determine the optimal cutoff values. Univariate and multivariate logistic regression analyses were used to identify independent predictors for postoperative AL separately in male and female. Before multivariate regression, the variance inflation factor (VIF) was calculated to detect the severity of multicollinearity. Independent variables with the greatest VIF were removed until the VIFs of all variables were less than 10. Two‐tailed *p* < 0.05 was considered statistically significant.

## 3 Results

### 3.1 Risk Factors for Postoperative AL in All Patients

**FIGURE 2:**
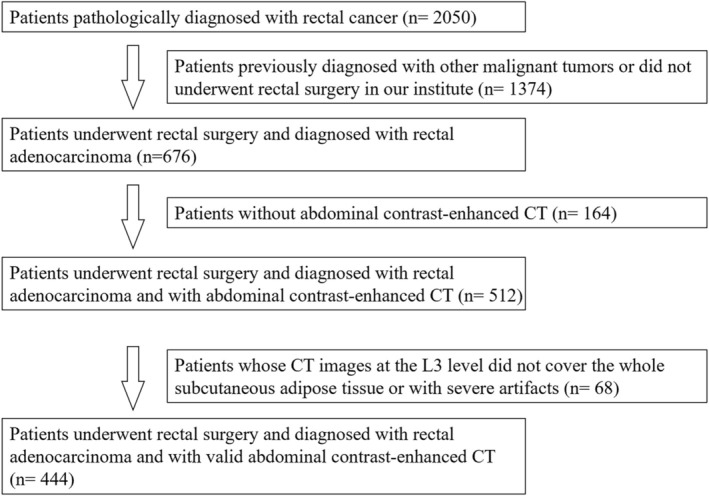
The flowchart of patient selection.

A total of 444 rectal cancer patients who underwent proctectomy were finally included in this study, including 21 patients (4.7%) in the AL group and 423 (95.3%) in the non‐AL group (Figure 2). According to the Clavien‐Dindo classification, out of the 21 patients in the AL group, 3 patients were grade II and were treated conservatively with antibiotics and nutritional support; 6 patients were grade IIIa and additional continuous flushing and drainage were performed; the remaining 12 patients were grade IIIb and relaparotomy under general anesthesia was required. Patients in the AL group were older than the non‐AL group (61.1 ± 12.2 years vs. 55.9 ± 11.2 years, *p* = 0.038). Patients in the AL group consumed more alcohol than the non‐AL group (0 (0, 125) mL vs. 0 (0, 0) mL, *p* = 0.038), and the cutoff value for alcohol consumption is > 75 mL (Table A1).

The pre‐ and post‐ surgical albumin in the AL group were significantly lower than those in the non‐AL group (*p* = 0.001 and < 0.001, respectively), and the cutoff value is ≤ 36.4 g/L and ≤ 29.5 g/L, respectively. Hypoalbuminemia was more common in the AL group (5 (25.0%) vs. 1 (0.2%), *p* < 0.001). WBC and neutrophil after surgery in 5 days were significantly higher in the AL group (*p* = 0.029 and 0.020, respectively), and the cutoff value is > 10.27*10^9^/L and > 8.05*10^9^/L, respectively (Tables A2 and A3). However, the body composition parameters of adipose tissue and skeletal muscle measured by CT images were not significantly different between both groups (Table A4).

### 3.2 Demographic Risk Factors for Postoperative AL in Different Gender

For male patients, there was no significant difference in demographic variables between the AL and the non‐AL groups. For female patients, AL patients were more likely to consume cigarettes and alcohol (*p* = 0.011 and < 0.001, respectively) with a longer duration time (*p* = 0.011 and < 0.001, respectively) and a higher smoking index (*p* = 0.010) and were more likely to have comorbid cardiovascular disease (*p* = 0.047) (Table 1).

**TABLE 1:** Demographic characteristics of patients in different genders.

	Male (*n* = 276)			Female (*n* = 168)		
	AL (*n* = 14)	Non‐AL (*n* = 262)	*p*	AL (*n* = 7)	Non‐AL (*n* = 161)	*p*
Age (years)	61.8 ± 12.9	57.2 ± 10.8	0.125	59.9 ± 11.5	53.9 ± 11.5	0.178
BMI (kg/m^2^)	23.4 (20.4, 26.1)	23 (21.4, 25)	0.839	22.2 ± 2	22.4 ± 3.2	0.836
BMI (categorical)			0.552			0.369
Underweight	1 (7.1%)	11 (4.2%)		1 (14.3%)	16 (9.9%)	
Normal	8 (57.1%)	186 (71.0%)		6 (85.7%)	112 (69.6%)	
Overweight	5 (35.7%)	59 (22.5%)		0 (0%)	31 (19.3%)	
Obese	0 (0%)	6 (2.3%)		0 (0%)	2 (1.2%)	
Cigarettes (per day)	0 (0, 20)	0 (0, 20)	0.938	0 (0, 0)	0 (0, 0)	0.011*
Duration (years)	0 (0, 16.3)	0 (0, 20)	0.442	0 (0, 0)	0 (0, 0)	0.010*
Smoking index	0 (0, 350)	0 (0, 400)	0.863	0 (0, 0)	0 (0, 0)	0.010*
Alcohol (ml/day)	0 (0, 212.5)	0 (0, 0)	0.353	0 (0, 100)	0 (0, 0)	< 0.001*
Duration (years)	0 (0, 12.5)	0 (0, 0)	0.652	0 (0, 20)	0 (0, 0)	< 0.001*
Hypertension	1 (7.1%)	49 (18.7%)	0.477	1 (14.3%)	16 (9.9%)	0.533
Diabetes	1 (7.1%)	19 (7.3%)	> 0.999	1 (14.3%)	11 (6.8%)	0.411
Heart diseases	0 (0%)	28 (10.7%)	0.374	2 (28.6%)	7 (4.3%)	0.047*
Radiotherapy	3 (21.4%)	36 (13.7%)	0.427	1 (14.3%)	13 (8.1%)	0.462
Chemotherapy	4 (28.6%)	53 (20.2%)	0.497	2 (28.6%)	23 (14.3%)	0.279

*Note:* Categorical variable is shown as number (percentage); continuous variable is shown as mean ± standard deviation or median (interquartile range). *p < 0.05.

### 3.3 Surgical and Pathological Risk Factors for Postoperative AL in Different Gender

For male patients, tumor location was a risk factor for postoperative AL (*p* = 0.011). Male patients with middle rectal cancer are more likely to develop AL after surgery. For female patients, the ASA score showed a significant difference between the AL and the non‐AL group (*p* = 0.038) (Table 2).

**TABLE 2:** Surgical and pathological parameters of patients in the AL and non‐AL groups separated by gender.

	Male (*n* = 276)			Female (*n* = 168)		
	AL (*n* = 14)	Non‐AL (*n* = 262)	*p*	AL (*n* = 7)	Non‐AL (*n* = 161)	*p*
Size (cm)	3.8 (2.3, 5.6)	3 (2.5, 4)	0.198	3 (2.5, 4)	3 (2.5, 3.5)	0.367
Location			0.011*			0.904
Upper	3 (21.4%)	90 (34.4%)		2 (28.6%)	47 (29.2%)	
Middle	11 (78.6%)	118 (45%)		4 (57.1%)	81 (50.3%)	
Lower	0 (0%)	54 (20.6%)		1 (14.3%)	33 (20.5%)	
Laparoscopy			> 0.999			> 0.999
No	0 (0%)	6 (2.3%)		0 (0%)	2 (1.2%)	
Yes	14 (100%)	256 (97.7%)		7 (100%)	159 (98.8%)	
Ileostomy			0.334			0.646
No	9 (64.3%)	201 (76.7%)		5 (71.4%)	125 (78.9%)	
Yes	5 (35.7%)	61 (23.3%)		2 (28.6%)	34 (21.1%)	
Tumor differentiation			0.177			0.400
Poor	2 (14.3%)	61 (23.3%)		3 (42.9%)	42 (26.1%)	
Moderate	10 (71.4%)	187 (71.4%)		3 (42.9%)	112 (69.6%)	
Well	2 (14.3%)	6 (2.3%)		1 (14.3%)	5 (3.1%)	
Other	0 (0%)	8 (3.1%)		0 (0%)	2 (1.2%)	
T stage			0.501			0.869
1	1 (7.1%)	16 (6.1%)		0 (0%)	6 (3.7%)	
2	4 (28.6%)	42 (16%)		2 (28.6%)	33 (20.5%)	
3	7 (50%)	127 (48.5%)		3 (42.9%)	73 (45.3%)	
4	2 (14.3%)	77 (29.4%)		2 (28.6%)	49 (30.4%)	
N stage			0.778			0.851
0	10 (71.4%)	164 (62.6%)		4 (57.1%)	84 (52.2%)	
1	2 (14.3%)	54 (20.6%)		2 (28.6%)	40 (24.8%)	
2	2 (14.3%)	44 (16.8%)		1 (14.3%)	37 (23%)	
M stage	0 (0%)	18 (6.9%)	0.609	0 (0%)	8 (5%)	> 0.999
Clinical stage			0.297			0.839
1	5 (35.7%)	48 (18.3%)		2 (28.6%)	34 (21.1%)	
2	5 (35.7%)	106 (40.5%)		2 (28.6%)	48 (29.8%)	
3	4 (28.6%)	91 (34.7%)		3 (42.9%)	71 (44.1%)	
4	0 (0%)	17 (6.5%)		0 (0) %	8 (5%)	
ASA score			0.477			0.038*
1	3 (21.4%)	24 (9.2%)		0 (0%)	20 (12.4%)	
2	9 (64.3%)	213 (81.3%)		5 (71.4%)	129 (80.1%)	
3	2 (14.3%)	24 (9.2%)		1 (14.3%)	12 (7.5%)	
4	0 (0%)	1 (0.4%)		1 (14.3%)	0 (0%)	

*Note:* Categorical variable is shown as number (percentage); continuous variable is shown as mean ± standard deviation or median (interquartile range). *p < 0.05.

### 3.4 Laboratory Test Risk Factors for Postoperative AL in Different Gender

Regardless of gender, the pre‐ and post‐surgical albumin in the AL group was significantly lower than those in the non‐AL group, and hypoalbuminemia as well as anemia were more common in the AL group (Table 3). For female patients, the post‐surgical WBC and neutrophil counts, neutrophil‐to‐lymphocyte ratio (NLR) and their pre‐post changes were significantly higher in the AL group. For male patients, pre‐surgical high NLR (> 3) was significantly associated with AL (Table 3).

**TABLE 3:** Laboratory test parameters of patients in the AL and non‐AL groups separated by gender.

	Male					Female				
	AL		Non‐AL		*p*	AL	Non‐AL			*p*
	Number	Value	Number	Value		Number	Value	Number	Value	
ALB_pre_ (g/L)	13	38.7 ± 4.9	261	41.2 ± 3.6	0.017*	7	37.2 ± 3.5	160	40.8 ± 4.0	0.018*
ALB_post_ (g/L)	14	30.3 ± 5.2	246	33.6 ± 3.8	< 0.001*	7	28.1 ± 3.1	146	31.9 ± 3.6	0.008*
ALB_change_ (g/L)	13	−8.4 ± 4.5	245	−7.6 ± 4.1	0.477	7	−9.1 ± 4.2	145	−9.0 ± 4.0	0.985
Hypoalbuminemia	3 (23.1%)		0 (0%)		< 0.001*	2 (28.6%)		1 (0.6%)		0.004*
WBC_pre_ (*10^9^/L)	13	6.5 (5, 7.5)	261	5.9 (4.9, 6.9)	0.515	7	6.6 (4.3, 7.0)	159	5.2 (4.4, 6.2)	0.383
WBC_post_ (*10^9^/L)	14	9.5 (7.2, 12)	249	9.1 (6.9, 11.2)	0.643	7	11.2 (10.3, 16.1)	149	7.6 (5.9, 10.6)	0.004*
WBC_change_ (*10^9^/L)	13	3.6 (1.2, 6.0)	248	3.1 (1.4, 5)	0.788	7	7.2 (3.8, 9.3)	147	2.5 (0.8, 4.6)	0.007*
Neu_pre_ (*10^9^/L)	13	4.0 (2.7, 5.4)	261	3.5 (2.7, 4.3)	0.329	7	3.9 (2.4, 4.8)	159	2.9 (2.3, 3.8)	0.259
Neu_post_ (*10^9^/L)	14	7.7 (5.9, 9.8)	249	7.2 (5.3, 9.4)	0.620	7	9.2 (8.9, 14.9)	149	6 (4.3, 8.6)	0.003*
Neu_change_ (*10^9^/L)	13	3.6 (1.9, 6.0)	248	3.6 (1.9, 5.9)	0.871	7	7.5 (4.2, 11.1)	147	2.9 (1.4, 5.2)	0.007*
Lym_pre_ (*10^9^/L)	13	1.4 (1.1, 1.7)	261	1.6 (1.3, 2.1)	0.156	7	1.3 (1.1, 2.0)	159	1.6 (1.3, 2)	0.321
Lym_post_ (*10^9^/L)	14	0.9 (0.5, 1.2)	249	1 (0.6, 1.3)	0.430	7	0.8 (0.4, 1.2)	149	1 (0.7, 1.2)	0.231
NLR_pre_	13	3.1 (1.8, 3.7)	261	2.1 (1.6, 2.9)	0.141	7	2.6 (1.6, 4.6)	159	1.9 (1.4, 2.5)	0.128
NLR_post_	14	9.8 (6.1, 13.3)	249	7.6 (5.3, 11.9)	0.236	7	12.3 (7.7, 37.3)	149	6.2 (4.3, 9.8)	0.006*
NLR_change_	13	6.9 (3.1, 12.1)	248	5.2 (3.0, 8.7)	0.476	7	9.9 (5.9, 35.1)	147	3.9 (2.3, 7.5)	0.013*
High NLR_pre_	7 (53.8%)		60 (23%)		0.019*	2 (28.6%)		21 (13.2%)		0.250
Hb_pre_ (g/L)	13	132 (116, 140)	260	134 (124.3, 145)	0.487	7	115 (98, 140)	159	120 (108, 128)	0.760
Anemia	4 (30.8%)		2 (0.8%)		< 0.001*	3 (42.9%)		2 (1.3%)		< 0.001*

*Note:* Categorical variable is shown as number (percentage); continuous variable is shown as mean ± standard deviation or median (interquartile range). *p < 0.05.

Abbreviations: ALB, albumin; Hb, hemoglobin; High NLR_pre_, NLR_pre_ > 3; Hypoalbuminemia, albumin < 35 g/L; Lym, lymphocyte; Neu, neutrophil; NLR, neutrophil‐to‐lymphocyte ratio; WBC, white blood cell.

### 3.5 Body Composition Risk Factors for Postoperative AL in Different Gender

As shown in Table 4, most of the body composition parameters of adipose tissue and skeletal muscle measured by CT images were significantly different between male and female genders. After being separately analyzed by gender, the IMAT mean CT value was significantly lower in the AL group (*p* = 0.029) in male patients. VSR was significantly associated with postoperative AL in both male and female patients, interestingly, showing gender differences in opposite directions. In male patients, VSR was a protective factor for the occurrence of AL. Male patients with higher VSR were less likely to develop postoperative AL. On the contrary, in female patients, VSR was positively associated with the occurrence of AL and served as a risk factor (Table 5). The cutoff value for VSR was ≤ 1.126 in male and > 0.828 in female.

**TABLE 4:** Body composition parameters of adipose tissue and skeletal muscle of patients in different genders.

	Male (*n* = 276)	Female (*n* = 168)	*p*
SAT mean value	−98.9 (−103.6, −92.9)	−106.9 (−110.5, −101.9)	< 0.001*
SAT area	101.6 (77.4, 134.2)	139.9 (106.1, 172)	< 0.001*
VAT mean value	−96.9 (−103, −89.3)	−97.7 (−102.8, −90.8)	0.727
VAT area	120.1 (59.5, 173.6)	74.8 (47.1, 112.7)	< 0.001*
IMAT mean value	−53.4 ± 6.0	−55.3 ± 5.6	0.001*
IMAT area	2.1 (1.1, 3.8)	2.8 (1.5, 4.4)	0.012*
SM mean value	41 (37, 45.4)	36.6 (31.5, 41)	< 0.001*
SM area	144.6 (131.9, 156.6)	100.2 (90.7, 110.4)	< 0.001*
SATI	35.2 (26.8, 47.8)	56 (41.9, 69.4)	< 0.001*
VATI	42.1 (21.5, 60.3)	30.2 (19.3, 45.8)	< 0.001*
IMATI	0.8 (0.4, 1.3)	1.2 (0.6, 1.8)	< 0.001*
SMI	144.6 (131.9, 156.6)	100.2 (90.7, 110.4)	< 0.001*
VSR	1.0 (0.7, 1.4)	0.5 (0.4, 0.7)	< 0.001*
SAT to SM ratio	0.7 (0.5, 0.9)	1.4 (1.1, 1.7)	< 0.001*
VAT to SM ratio	0.8 (0.4, 1.2)	0.7 (0.5, 1.1)	0.625
IMAT to SM ratio	0.02 (0.01, 0.03)	0.03 (0.01, 0.05)	< 0.001*

*Note:* categorical variable is shown as number (percentage); continuous variable is shown as mean ± standard deviation or median (interquartile range). *p < 0.05.

Abbreviations: IMAT, intramuscular adipose tissue; IMATI, IMAT index; SAT, subcutaneous adipose tissue; SATI, SAT index; SM, skeletal muscle; SMI, SM index; VAT, visceral adipose tissue; VATI, VAT index; VSR, visceral to subcutaneous adipose tissue ratio.

**TABLE 5:** Body composition parameters of adipose tissue and skeletal muscle of patients in the AL and non‐AL groups separated by gender.

	Male (*n* = 276)			Female (*n* = 168)		
	AL (*n* = 14)	Non‐AL (*n* = 262)	*p*	AL (*n* = 7)	Non‐AL (*n* = 161)	*p*
SAT mean value	−95.9 (−101.5, −92.6)	−98.9 (−103.6, −92.8)	0.425	−103.3 (−111.8, −94)	−106.9 (−110.5, −102.2)	0.468
SAT area	99.5 (56.6, 161.3)	101.7 (77.6, 133)	0.966	153 (81.6, 170.7)	139.7 (106.4, 172.7)	0.778
VAT mean value	−96.6 (−100.9, −87.7)	−96.9 (−103, −89.5)	0.470	−89.5 (−104.9, −88.4)	−97.8 (−102.8, −91.1)	0.448
VAT area	108.8 (68.7, 166.8)	120.6 (59.4, 174.8)	0.960	95.3 (43.2, 135.1)	74.6 (47.1, 112.6)	0.871
IMAT mean value	−56.8 ± 4.3	−53.2 ± 6.1	0.029*	−51.9 ± 5.2	−55.4 ± 5.6	0.101
IMAT area	3.1 (2, 4.5)	2.1 (1.1, 3.8)	0.111	2.3 (0.5, 5.6)	2.8 (1.5, 4.4)	0.736
SM mean value	39.5 (36.1, 45.2)	41.1 (37, 45.5)	0.633	37.5 ± 9.5	36 ± 7.3	0.591
SM area	141.1 ± 14.5	145.3 ± 20.8	0.454	107.5 ± 23	101 ± 16.1	0.308
SATI	35.5 (19.7, 55.4)	35.2 (27.1, 46.8)	0.989	59.8 (27.3, 65.9)	55.3 (41.9, 69.6)	0.984
VATI	38.1 (23.3, 55.7)	42.5 (21.5, 60.4)	0.891	32.6 (14.4, 52.1)	30 (19.3, 45.8)	0.972
IMATI	1.1 (0.7, 1.6)	0.7 (0.4, 1.3)	0.118	0.9 (0.2, 1.9)	1.2 (0.6, 1.8)	0.648
SMI	49.1 ± 5.1	50.8 ± 7.1	0.381	39.6 ± 6.5	40.1 ± 6	0.849
VSR^a^	0.7 (0.5, 1.1)	1 (0.7, 1.4)	0.021*	1.5 (0.5, 1.5)	0.5 (0.4, 0.7)	0.008*
SAT to SM ratio	0.7 ± 0.3	0.7 ± 0.3	0.964	1.5 (0.8–1.7)	1.4 (1.1, 1.7)	0.815
VAT to SM ratio	0.8 (0.5, 1.1)	0.8 (0.4, 1.2)	0.981	0.9 (0.3–1.2)	0.7 (0.5, 1.1)	0.959
IMAT to SM ratio	0.02 (0.02–0.03)	0.01 (0.01–0.03)	0.108	0.02 (0.01, 0.05)	0.03 (0.01, 0.05)	0.724

*Note:* continuous variable is shown as mean ± standard deviation or median (interquartile range). *p < 0.05.

Abbreviations: IMAT, intramuscular adipose tissue; IMATI, IMAT index; SAT, subcutaneous adipose tissue; SATI, SAT index; SM, skeletal muscle; SMI, SM index; VAT, visceral adipose tissue; VATI, VAT index; VSR, visceral to subcutaneous adipose tissue ratio.

^a^ The cutoff value is ≤ 1.126 in male patients and > 0.828 in female patients.

### 3.6 Independent Risk Factors for Postoperative AL in Different Gender

Due to multicollinearity, some variables, especially laboratory test results which were highly correlated with each other, were removed from multivariate logistic regression. The results of multivariate regression showed that after controlling for confounding factors, VSR remained an independent protective/risk factor for postoperative AL in rectal cancer (Table 6). In male patients, VSR was negatively associated with AL with an odds ratio (OR) of 0.1 (95% CI: 0–0.9) (*p* = 0.041), while in female patients, VSR was positively associated with AL (OR: 39.1 (95% CI: 1.1– > 100), *p* = 0.045). Other independent risk factors for AL in males included pre‐surgical high NLR (OR: 4.7 (95% CI: 1.2–18.4), *p* = 0.026) and anemia (OR: 44.2 (95% CI: 5.7–345.3), *p* < 0.001); in females, these included pre‐post change of NLR (OR: 1.1 (95% CI: 1.0–1.2), *p* = 0.049) (Table 6).

**TABLE 6:** Univariate and multivariate logistic regression for risk factors predicting postoperative AL in different genders.

		Univariate analysis		Multivariate analysis	
		OR (95% CI)	*p*	OR (95% CI)	*p*
Male	Tumor location	0.5 (0.2, 1.0)	0.037*		
	ALB_pre_ (g/L)	0.8 (0.7, 1.0)	0.018*		
	High NLR_pre_	3.9 (1.3, 12.1)	0.018*	4.7 (1.2, 18.4)	0.026*
	Anemia	57.3 (9.3, > 100)	< 0.001*	44.2 (5.7, 345.3)	< 0.001*
	IMAT mean value	0.9 (0.8, 1)	0.030*		
	VSR	0.2 (0.1, 0.9)	0.030*	0.1 (0, 0.9)	0.041*
Female	Heart disease	8.8 (1.4, 53.6)	0.018*		
	Smoking index	1.01 (1.00, 1.02)	0.030*		
	ASA score	5.7 (1.5, 21.9)	0.011*		
	ALB_pre_ (g/L)	0.8 (0.7, 1)	0.023*		
	NLR_change_	1.1 (1, 1.2)	0.002*	1.1 (1.0, 1.2)	0.049*
	Anemia	58.9 (7.6, > 100)	< 0.001*		
	VSR	91.8 (9.5, > 100)	< 0.001*	39.1 (1.1, > 100)	0.045*

*Note:* *p < 0.05.

Abbreviations: 95% CI, 95% confidence interval; ALB, albumin; High NLR_pre_, NLR_pre_ > 3; IMAT, intramuscular adipose tissue; NLR, neutrophil‐to‐lymphocyte ratio; OR, odds ratio; VSR, visceral to subcutaneous adipose tissue ratio.

## 4 Discussion

Our study revealed that the VSR measured by CT images was gender‐dependently associated with the postoperative AL in rectal cancer. In male patients, VSR was an independent protective factor for the occurrence of AL, while in female patients, VSR served as an independent risk factor.

The overall incidence of postoperative AL in our cohort was 4.7% (5.1% in male population and 4.2% in female population), which was relatively low compared with the previously reported incidence of 5%–19% [14]. Due to the lack of a generally accepted definition of postoperative AL in rectal cancer, various definitions were adopted by previous studies [19]; the relatively low AL rate in our cohort might partly be attributed to the strict definition of AL in our study. AL increases postoperative morbidities and mortalities, as well as risks for reoperation, permanent stoma formation, and local recurrence; therefore, it negatively affects patients' short‐term and long‐term outcomes [7, 14, 20]. Identifying risk factors for AL allows early prevention and timely detection and intervention.

There is increasing evidence that body composition parameters measured by CT images might correlate with postoperative complications in colorectal cancer [21]; however, their impacts on the occurrence of postoperative AL in rectal cancer were inconclusive [10, 22, 23]. In accordance with our findings, previous studies found no correlations between body composition parameters (including VAT area and sarcopenia) and AL after rectal cancer surgery [10, 22, 23]. On the contrary, a meta‐analysis showed that patients with higher VAT area have a higher incidence of AL in both colon and rectal cancer, although the correlation was more prominent in colon cancer [24]. Our present study manifested that VAT area, as well as other body composition parameters, such as areas of SAT, SM, and IMAT, VATI, SATI, SMI, and IMATI, were not associated with AL in rectal cancer patients, neither in all patients nor in different gender subgroups. Instead, we discovered that VSR was significantly associated with AL in both male and female patients. VSR, the relative proportion of VAT area in relation to SAT area, is a crucial parameter reflecting abdominal adipose tissue distribution. High VSR, also termed visceral adiposity [4], is associated with an increased risk of cancer development, possibly owing to the resultant chronic systemic inflammation that alters metabolic environments and ultimately mediates obesity‐related carcinogenesis [25]. High VSR was acknowledged as a potential predictor for postoperative overall complications, recurrence, and poor survival in rectal cancer [26, 27, 28]. Similar to Kaess's findings [29], in our cohort, male patients' VSR was significantly higher than that of female patients. Therefore, it is reasonable to analyze patients separately by gender. In the present study, high VSR was an independent risk factor for postoperative AL in female patients, which was in accordance with previous studies [26, 27, 28]; however, high VSR turned out to be an independent protective factor for AL in male patients. It is generally accepted that the narrow male pelvis is technically difficult to perform rectal surgery [14] and excessive visceral fat might make it difficult to accurately identify lesions, vessels, and lymph nodes, especially for the laparoscopic procedure. Our findings in male patients seemed controversial. Similar to our results in male patients, another research with the majority being male (71%) indicated that visceral adiposity may be a protective factor for postoperative AL in rectal cancer [9] and this so‐called obesity paradox effect was attributed to the mechanical protection of the anastomosis by the surrounding adipose tissue. In our cohort, the median and interquartile range of VSR in male patients was 1.0 (0.7, 1.4) (Table 4), and 9 patients in the male AL group had VSR less than 1, with 6 patients having extremely low VSR less than 0.7. Whether there might be a similar J‐shaped relationship between VSR and AL, as observed in other diseases with moderate body fat for better prognosis [9] or whether this association between low VSR and AL observed in our male cohort is a result of mixed effects of metabolism and nutrition is still unknown. Further large prospective studies are warranted to validate this controversial result between VSR and AL. Previous studies seldom analyzed the relationship between body composition parameters and postoperative AL in rectal cancer separately by gender. This present study focused on body composition parameters derived from contrast‐enhanced CT, a commonly used imaging modality for rectal cancer staging, in different genders and found significant association between VSR and postoperative AL with gender differences in opposite directions.

The correlation between age and postoperative AL in rectal cancer is still inconclusive. Similar to a previous study, our study found that older age was positively associated with AL in all patients [30]. After separately analyzing by gender, patients in the AL group were still older than the non‐AL group in both male and female patients, although without statistical significance. However, some studies showed that increased age seemed to be a protective factor for AL [31, 32], while others showed a similar incidence of AL in younger and elderly rectal cancer patients [33]. Other risk factors for AL included cigarette and alcohol consumption (both daily amounts and durations), comorbid heart diseases, and ASA score, which were in accordance with previous studies [34, 35, 36, 37]; however, only in female patients in our cohort. Although smoking and alcohol abuse are acknowledged as risk factors for AL in colorectal surgery by previous studies, perhaps due to the impaired wound healing associated with ischemia and poor nutrition [34, 38], however, the trends were observed only in female patients in our cohort. This might be explained by the high prevalence of cigarette and alcohol consumption in male patients and the significant differences in the prevalence between male and female patients in our cohort (40.9% versus 1.8% for cigarette consumption and 22.5% versus 1.8% for alcohol consumption). Owing to the relatively small sample size in our AL group, further prospective investigations with a larger sample size are needed.

Tumor location was another risk factor for postoperative AL in rectal cancer in male and all patients in our cohort. AL appeared to occur more frequently in the cancers in the middle third of the rectum, not in agreement with other studies, which showed that the distal site of the tumor was one of the most significant risk factors for AL [7, 14, 38]. This discordance might be explained by the small sample size in lower rectal cancer in our cohort, which might be the result of the strict criteria for surgical candidate selection in lower rectal cancer in our institute. Another possible explanation might be that lower rectal cancer is one of the indications for ileostomy after rectal cancer surgery in our institute (other indications include severe malnutrition, complicated preoperative comorbidities, advanced clinical T stage, large tumor size, significant intraoperative blood loss and prolonged operative duration). According to a previous meta‐analysis [39], ileostomy might be associated with a lower AL rate after rectal cancer surgery, which might partly explain the fact that in our study, lower tumor location was not associated with the incidence of AL. Similar to a previous study showing that ileostomy decreased the AL severity and relaparotomy rate [15], in our study, out of the 12 patients with Clavien‐Dindo classification grade IIIb requiring relaparotomy, 10 patients were without deviating ileostomy. Therefore, ileostomy might also partly explain the relatively low AL rate in our cohort.

In accordance with previous studies [14, 36], some laboratory test indicators for poor nutrition, including pre‐ and post‐surgical low albumin level, hypoalbuminemia, and anemia, were significantly associated with AL regardless of gender. For female and all patients, the post‐surgical WBC and neutrophil counts, as well as post‐surgical NLR and its pre‐post change, all reflecting elevated systemic inflammation after surgery, were higher in the AL group with statistical significance. Previous studies suggested that preoperative high NLR, a biomarker for systemic inflammation, was an independent predictor for poor prognosis in colorectal cancer [40, 41]. Our present study found an association between preoperative high NLR and postoperative AL in male and all patients. A similar trend was observed in female patients, although without statistical significance; this might be attributed to the small sample size in the AL group for female patients.

Our study has several limitations. First, due to the relatively low incidence of AL in our cohort, the sample size in the AL group was relatively small. The relatively low AL rate in our cohort might partly be attributed to the strict definition of AL in our study, and some cases with subclinical AL not requiring medical intervention might be missed, especially in patients with ileostomy. Nevertheless, it is the AL requiring medical intervention that really matters in clinical scenarios. Second, body composition parameters were measured using a single cross‐sectional CT image at the L3 level instead of volumetric measurement. Although volumetric measurement of the total abdominal adipose tissue and skeletal muscle compartments might represent body composition more comprehensively, a previous study manifested that single‐slice measurements of SAT, VAT, and SM at different lumbar levels were strongly associated with volumetric measurements and were simple and convenient [42]. Third, due to the retrospective nature of this study, some potential biomarkers for postoperative AL, such as CRP and PCT [14] were not available in most of our patients; as a result, the predictive values of these factors were not discussed in this study. Future prospective studies with a larger sample size are warranted to further explore risk factors for postoperative AL in rectal cancer.

In conclusion, the VSR measured by CT images is an independent predictor for postoperative AL in patients with rectal cancer; however, it shows gender differences in opposite directions, serving as a protective factor in males, while as a risk factor in females. Further prospective investigations with a larger sample size are needed.

## Author Contributions

**Yan Luo:** formal analysis (equal), funding acquisition (equal), writing – original draft (equal), writing – review and editing (equal). **Jian Liu:** data curation (supporting), writing – original draft (equal), writing – review and editing (equal). **Jiong Huang:** data curation (equal), writing – review and editing (equal). **Liya Ma:** conceptualization (equal), data curation (equal), formal analysis (equal), supervision (lead), writing – original draft (equal), writing – review and editing (equal). **Zhen Li:** conceptualization (equal), funding acquisition (equal), supervision (equal), writing – review and editing (equal).

## Ethics Statement

This retrospective study was approved by the Ethics Committee of Tongji Hospital, Tongji Medical College, Huazhong University of Science and Technology (approval number: TJ‐IRB202420206). Informed patient consent was waived.

## Conflicts of Interest

The authors declare no conflicts of interest.

## Data Availability

The datasets used and/or analyzed during the current study are available from the corresponding author on reasonable request.

## Appendix A

**TABLE A1:** Demographic characteristics of patients.

	AL (*n* = 21)	Non‐AL (*n* = 423)	*p*
Sex (male)	14 (66.7%)	262 (61.9%)	0.663
Age (years)	61.1 ± 12.2	55.9 ± 11.2	0.038*
BMI (kg/m^2^)	22.8 (21.1, 25)	22.8 (21, 24.8)	0.880
BMI (categorical)			0.767
Underweight	2 (9.5%)	27 (6.4%)	
Normal	14 (66.7%)	298 (70.4%)	
Overweight	5 (23.8%)	90 (21.3%)	
Obese	0 (0%)	8 (1.9%)	
Cigarettes per day	0 (0, 15)	0 (0, 5)	0.645
Duration (years)	0 (0, 12.5)	0 (0, 5)	0.996
Smoking index	0 (0, 200)	0 (0, 0)	0.670
Alcohol (ml/day)	0 (0, 125)	0 (0, 0)	0.038*
Duration (years)	0 (0, 15)	0 (0, 0)	0.074
Hypertension	2 (9.5%)	65 (15.4%)	0.361
Diabetes	2 (9.5%)	30 (7.1%)	0.657
Heart disease	2 (9.5%)	35 (8.3%)	0.691
Radiotherapy	4 (19%)	49 (11.6%)	0.298
Chemotherapy	6 (28.6%)	76 (18%)	0.247

*Note:* categorical variable is shown as number (percentage); continuous variable is shown as mean ± standard deviation or median (interquartile range). **p* < 0.05.

**TABLE A2:** Surgical and pathological parameters of patients in the AL and non‐AL groups of all patients.

	AL (*n* = 21)	Non‐AL (*n* = 423)	*p*
Size (cm)	3 (2.5–5)	3 (2.5–4)	0.123
Location			0.044*
Upper	5 (23.8%)	137 (32.4%)	
Middle	15 (71.4%)	199 (47.0%)	
Lower	1 (4.8%)	87 (20.6%)	
Laparoscopy			> 0.999
No	0 (0%)	8 (1.9%)	
Yes	21 (100%)	415 (98.1%)	
Ileostomy			0.286
No	14 (66.7%)	328 (77.5%)	
Yes	7 (33.3%)	95 (22.5%)	
Tumor differentiation			0.110
Poor	5 (23.8%)	103 (24.3%)	
Moderate	13 (61.9%)	299 (70.7%)	
Well	3 (14.3%)	11 (2.6%)	
Other	0 (0%)	10 (2.4%)	
T stage			0.570
1	1 (4.8%)	22 (5.2%)	
2	6 (28.6%)	75 (17.7%)	
3	10 (47.6%)	200 (47.3%)	
4	4 (19%)	126 (29.8%)	
N stage			0.749
0	14 (66.7%)	248 (58.6%)	
1	4 (19%)	94 (22.2%)	
2	3 (14.3%)	81 (19.1%)	
M stage	0 (0%)	26 (6.1%)	0.625
Clinical stage			0.236
1	7 (33.3%)	82 (19.4%)	
2	7 (33.3%)	154 (36.4%)	
3	7 (33.3%)	162 (38.3%)	
4	0 (0%)	25 (5.9%)	
ASA score			0.182
1	3 (14.3%)	44 (10.4%)	
2	14 (66.7%)	342 (80.9%)	
3	3 (14.3%)	36 (8.5%)	
4	1 (4.8%)	1 (0.2%)	

*Note:* categorical variable is shown as number (percentage); continuous variable is shown as mean ± standard deviation or median (interquartile range). **p* < 0.05.

**TABLE A3:** Laboratory test parameters of patients in the AL and non‐AL groups of all patients.

	AL group		Non‐AL group		*p*
	No.	Value	No.	Value	
ALB_pre_ (g/L)	20	38.2 ± 4.4	421	41.1 ± 3.8	0.001*
ALB_post_ (g/L)	21	29.5 ± 4.7	392	33 ± 3.8	0.000*
ALB_change_ (g/L)	20	8.6 ± 4.3	390	8.1 ± 4.1	0.584
Hypoalbuminemia	5 (25%)		1 (0.2%)		< 0.001*
WBC_pre_ (*10^9^/L)	20	6.5 (4.7, 7.2)	420	5.6 (4.6, 6.7)	0.252
WBC_post_ (*10^9^/L)	21	10.4 (7.9, 13.7)	398	8.6 (6.5, 11)	0.029*
WBC_change_ (*10^9^/L)	20	4 (1.7, 8.5)	395	2.8 (1, 4.8)	0.058
Neu_pre_ (*10^9^/L)	20	3.9 (2.6, 5.3)	420	3.3 (2.5, 4.2)	0.161
Neu_post_ (*10^9^/L)	21	8.6 (6.2, 11.8)	398	6.9 (5, 9.1)	0.020*
Neu_change_ (*10^9^/L)	20	4.5 (2.6, 9.1)	395	3.4 (1.6, 5.5)	0.058
Lym_pre_ (*10^9^/L)	20	1.4 (1.1, 1.7)	420	1.6 (1.3, 2)	0.081
Lym_post_ (*10^9^/L)	21	0.9 (0.5, 1.2)	398	1 (0.7, 1.3)	0.177
NLR_pre_	20	2.7 (1.8, 3.7)	420	2 (1.5, 2.7)	0.036*
NLR_post_	21	10 (6.8, 18.1)	398	7.1 (4.9, 10.7)	0.007*
NLR_change_	20	7.5 (3.9, 17.9)	395	4.8 (2.8, 8.3)	0.032*
High NLR_pre_	9 (45%)		81 (19.3%)		0.010*
Hb (g/L)	20	130 (112.5, 138.8)	419	129 (115, 140)	0.826
Anemia	7 (35%)		4 (1%)		< 0.001*

*Note:* Categorical variable is shown as number (percentage); continuous variable is shown as mean ± standard deviation or median (interquartile range). **p* < 0.05.

Abbreviations: ALB, albumin; Hb, hemoglobin; Lym, lymphocyte; Neu, neutrophil; NLR, neutrophil‐to‐lymphocyte ratio; WBC, white blood cell.

**TABLE A4:** Body composition parameters of fat tissue and skeletal muscle of patients in the AL and non‐AL groups of all patients.

	AL (*n* = 21)	Non‐AL (*n* = 423)	*p*
SAT mean value	−97.8 (−105.7, −93.2)	−102.1 (−107.4, −95.3)	0.182
SAT area	107.5 (75.6, 161.8)	116.5 (86.4, 151)	0.987
VAT mean value	−96.5 (−101.9, −88.2)	−97.2 (−102.9, −90.1)	0.305
VAT area	106.1 (53.5, 139.1)	97.9 (52.8, 149.8)	0.896
IMAT mean value	−55.2 ± 5.1	−54.1 ± 6	0.402
IMAT area	3.1 (2–4.5)	2.4 (1.2–4.1)	0.327
SM mean value	39.2 ± 7.1	39 ± 7.5	0.902
SM area	139.5 (110.9, 150.8)	130.3 (104.1, 148.6)	0.683
SATI	37.2 (26.9, 60)	41.7 (31.1, 56.8)	0.762
VATI	37.7 (19.7, 51.7)	36 (20.3, 53.9)	0.944
IMATI	1 (0.7, 1.6)	0.9 (0.4, 1.5)	0.433
SMI	45.9 ± 7.1	46.7 ± 8.5	0.684
VSR	0.8 (0.5, 1.1)	0.8 (0.5, 1.2)	0.645
SAT to SM ratio	0.8 (0.5, 1.3)	0.9 (0.6, 1.3)	0.702
VAT to SM ratio	0.8 (0.5, 1.2)	0.8 (0.5, 1.2)	0.942
IMAT to SM ratio	0 (0, 0)	0 (0, 0)	0.440

*Note:* categorical variable is shown as number (percentage); continuous variable is shown as mean ± standard deviation or median (interquartile range).

Abbreviations: IMAT, intramuscular adipose tissue; IMATI, IMAT index; SAT, subcutaneous adipose tissue; SATI, SAT index; SM, skeletal muscle; SMI, SM index; VAT, visceral adipose tissue; VATI, VAT index; VSR, visceral to subcutaneous adipose tissue ratio.
